# The Astrovirus Capsid: A Review

**DOI:** 10.3390/v9010015

**Published:** 2017-01-19

**Authors:** Carlos F. Arias, Rebecca M. DuBois

**Affiliations:** 1 Departamento de Genética del Desarrollo y Fisiología Molecular, Instituto de Biotecnología, Universidad Nacional Autónoma de México, Cuernavaca, Morelos 62210, Mexico; arias@ibt.unam.mx; 2 Department of Biomolecular Engineering, University of California, Santa Cruz, CA 95064, USA

**Keywords:** astrovirus, capsid, antigen, structure, virus entry, virus exit

## Abstract

Astroviruses are enterically transmitted viruses that cause infections in mammalian and avian species. Astroviruses are nonenveloped, icosahedral viruses comprised of a capsid protein shell and a positive-sense, single-stranded RNA genome. The capsid protein undergoes dramatic proteolytic processing both inside and outside of the host cell, resulting in a coordinated maturation process that affects cellular localization, virus structure, and infectivity. After maturation, the capsid protein controls the initial phases of virus infection, including virus attachment, endocytosis, and genome release into the host cell. The astrovirus capsid is the target of host antibodies including virus-neutralizing antibodies. The capsid protein also mediates the binding of host complement proteins and inhibits complement activation. Here, we will review our knowledge on the astrovirus capsid protein (CP), with particular attention to the recent structural, biochemical, and virological studies that have advanced our understanding of the astrovirus life cycle.

## 1. Introduction

The family *Astroviridae* is divided into two genera: genus *Mamastrovirus* including viruses that infect mammals, and genus *Avastrovirus* including viruses that infect avian species. Astrovirus infections in birds cause diverse pathologies including enteritis, hepatitis, and nephritis. Astrovirus infections in mammals typically cause gastroenteritis and in rare cases cause neurological syndromes and encephalitis. Human astroviruses (HAstV) are recognized as a leading cause of childhood viral gastroenteritis, with eight canonical serotypes (HAstV-1 to HAstV-8) and several noncanonical human genogroups.

Astroviruses are nonenveloped, T = 3 icosahedral viruses with a positive-sense, single-stranded RNA genome of 6–8 kb (reviewed in [1,2]). The astrovirus genome contains 5′ and 3′ untranslated regions and three open reading frames (ORFs). The two ORFs at the 5′-end of the genome, ORF1a and ORF1b, encode nonstructural polyproteins nsp1a and nsp1ab, which are proteolytically processed into smaller proteins including an RNA-dependent RNA polymerase, a serine protease, a viral genome-linked protein (VPg), and several other proteins with unknown functions. The third ORF at the 3′-end of the genome, ORF2, encodes the astrovirus capsid protein (CP). In this review, we focus on the astrovirus CP: its assembly into virus particles, its processing, its structure, its interactions with host antibodies and complement, and its entry into cells.

## 2. Capsid Assembly and Proteolytic Processing

**Figure 1:**
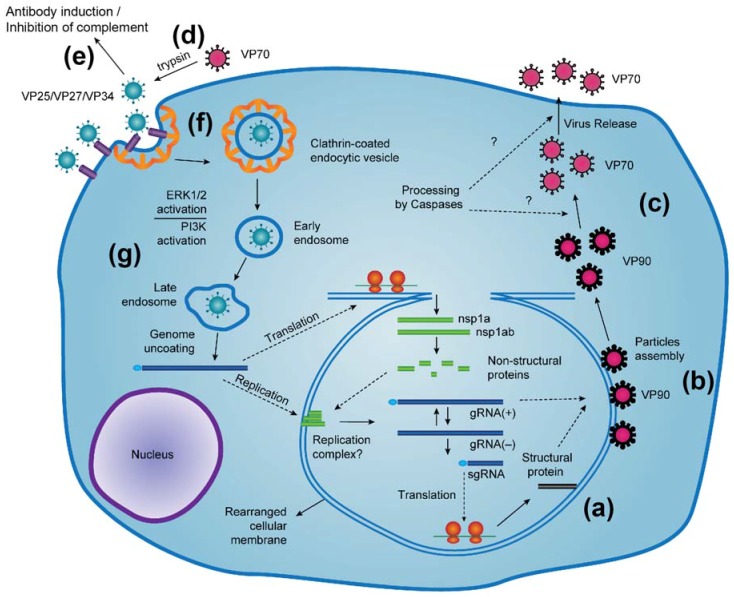
Replication cycle of human astroviruses (adapted from [2]). (**a**) Synthesis of the 90 kDa human astroviruses (HAstV) capsid protein (VP90) from viral subgenomic RNA (sgRNA); (**b**) Assembly of VP90 proteins (180 copies) with viral genomic RNA into HAstV particles; (**c**) Caspase-mediated cleavage of VP90 C-termini to form VP70 and subsequent release of immature HAstV particles; (**d**) Extracellular protease cleavage of immature HAstV particles to form mature, infectious HAstV particles. In cell culture, trypsin is used to produce mature, infectious HAstV particles; (**e**) Extracellular HAstV particles induce host antibody production and inhibit host complement activation; (**f**) Attachment and clathrin-dependent endocytosis of HAstV particles; (**g**) Uncoating of the virus genome in the late endosome. Extracellular signal-regulated kinase (ERK1/2) and phosphoinositide 3-kinase (PI3K) are activated during HAstV binding or entry into the cell.

**Figure 2:**
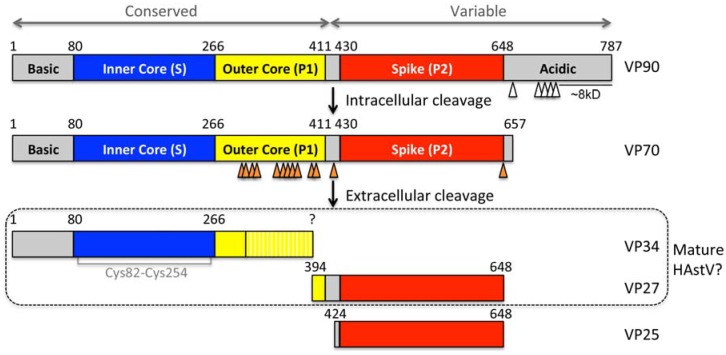
Schematic of HAstV-1 capsid protein (CP) domain structure and proteolytic processing (adapted from [14]). The CP is colored by structural domains, with the inner core domain in blue, the outer core domain in yellow, and the spike domain in red. Putative caspase and trypsin cleavage sites are specified with white and orange arrows, respectively. Putative trypsin cleavage sites (arginine and lysine residues) are noted only in the outer core region. The yellow hatched region in VP34 represents a region that is cleaved at multiple sites by trypsin but likely remains structured as part of the core domain in the mature HAstV virion. Putative caspase cleavage sites were previously predicted [12].

The astrovirus CP is synthesized from the viral subgenomic RNA (sgRNA) and is 72–90 kDa [3,4,5,6] (Figure 1a). In general, avian astrovirus CPs are shorter (72–80 kDa) than mammalian astrovirus CPs (80–90 kDa). The N-terminal half of the CP is more conserved among all astroviruses, whereas the C-terminal half shows high sequence variability (Figure 2). For HAstV-1–8, the CPs have overall amino acid sequence identities of 64%–85%, where the N-terminal halves are 84%–97% identical and the C-terminal halves are 39%–77% identical. All astroviruses CPs contain a region of basic amino acids (arginines and lysines) at the N-terminus that is thought to interact with the genomic RNA inside the virion [7] (Figure 2).

In HAstV-infected Caco-2 cells (human colon carcinoma cells, the model cell line for HAstV infection), the 90 kDa HAstV capsid protein (VP90) is produced and assembles into viral particles [8,9] (Figure 1b and Figure 2). Analysis of the HAstV-8-infected cells by density ultracentrifugation shows that intracellular viral particles comprised of VP90 are present in both membrane and membrane-free fractions, suggesting that viral progeny may assemble in membranous structures [9]. Indeed, using immunoelectron microscopy, viral particles are observed in clusters and associated at the edges of membranous vesicles [9]. VP90 is then cleaved at the C-terminus by cellular caspases to produce a 70 kDa protein (VP70) [10,11,12] (Figure 1c and Figure 2). This cleavage occurs through several intermediate cleavage events, consistent with several putative caspase cleavage sites identified in the CP acidic domain at the C-termini of many astrovirus capsid proteins [12] (Figure 2). Many cellular caspases are activated during HAstV infection, and caspase inhibitors were found to block the cleavage of VP90 to VP70 [12]. Studies with RNA interference reveal that knocking down caspases-3 and -9, but not caspase-8, blocked cleavage of the capsid protein in cells [10]. It is not known if the released C-terminal fragments of the astrovirus capsid protein have any function inside the host cell, although it is interesting to note that the ~8 kDa C-terminal region of the HAstV capsid protein (Figure 2) has high sequence conservation between serotypes HAstV-1–8 (~50% identity) and secondary structure prediction programs predict that this region is helical in structure [13].

Caspase cleavage is required for HAstV release from cells, since the presence of caspase inhibitors drastically reduces the release of virus particles [10,12] (Figure 1c). While the mechanism of virus release is unknown, it does appear to occur in the absence of cell death [10]. Instead, a non-lytic mechanism is thought to occur, perhaps by membrane destabilization or via membrane vesicles, although studies to characterize the mechanism of virus exit have yet to be performed.

Once released outside of the host cell, immature HAstV particles comprised of VP70 are further processed by extracellular proteases to produce mature, infectious HAstV particles (Figure 1d). In cell culture, HAstV maturation is achieved by proteolysis with trypsin, which increases viral infectivity by 10^5^-fold [11,15,16]. For the HAstV-8 CP, trypsin digestion results in the cleavage of VP70 into three predominant protein fragments: VP34, VP27, and VP25 [11]. Similar results were observed for the HAstV-1 and HAstV-2 CPs [15,17] (Figure 2), although the size and number of fragments observed appears to be dependent on the HAstV serotype (reviewed in [18]). N-terminal sequencing studies have identified HAstV-8 CP residues 394 and 424 at the N-termini of VP27 and VP25, respectively, revealing that two versions of fragments encompassing the capsid’s variable C-terminal region are formed [11]. VP34 encompasses the conserved N-terminal region of the capsid protein. Although trypsin is used for maturation in cell culture, it is possible that other proteases may play a role in HAstV maturation and infectivity in vivo.

## 3. Capsid Structure

In 1975, astroviruses were first visualized by negative-stain electron microscopy in stool samples of children with diarrhea and vomiting [19,20]. Virus particles were observed to have a distinct star-like appearance (*astron* means star in Greek). Infectious HAstV particles grown in cell culture and visualized by electron microscopy were observed to be ~41 nm in diameter and form spherical particles studded with spikes [21].

**Figure 3:**
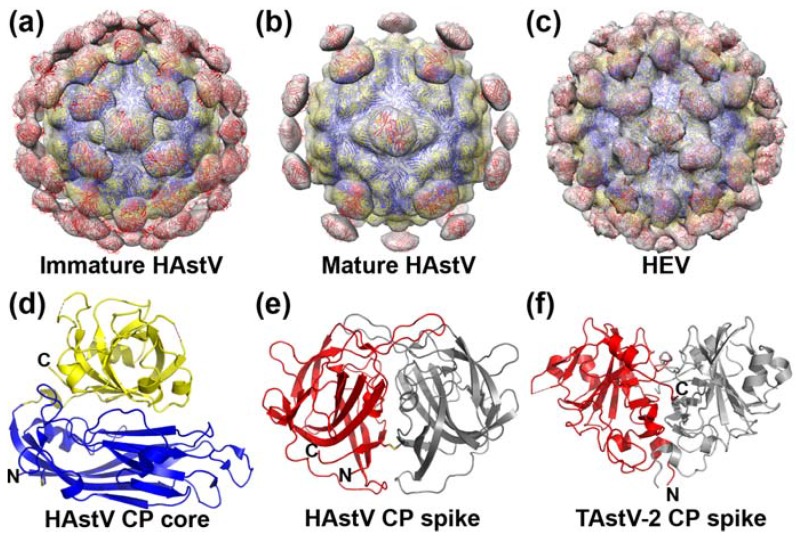
CryoEM and crystal structures of the astrovirus CP. (**a**) Immature T = 3 HAstV model made by fitting CP core and spike crystal structures into the immature HAstV virion cryo-EM map [22]; (**b**) Mature T = 3 HAstV model made by fitting CP core and spike crystal structures into the mature (trypsin-digested) HAstV virion cryo-EM map [22]; (**c**) Hepatitis E Virus (HEV) CP structure fit into the HEV T = 3 virus-like particle cryo-EM maps [25]; (**d**) Crystal structure of the HAstV-1 CP core domain [14]; (**e**) Crystal structure of the dimeric HAstV-1 CP spike domain [14]; (**f**) Crystal structure of the dimeric turkey astrovirus 2 (TAstV-2) CP spike domain [26]. All CP domains are colored as in Figure 2.

In the past five years, several exciting new structural studies have revealed high-resolution information about the astrovirus capsid protein and its assembly into immature and mature HAstV particles. First, the structures of immature (VP70) and mature, infectious (trypsin-digested) HAstV particles were resolved to moderate ~25 Å resolution by cryo-electron microscopy (cryo-EM) [22]. Both immature and mature HAstV particles are ~43 nm in diameter and have an inner, continuous core layer and an outer layer comprised of globular spikes (Figure 3a,b). The inner layer is ~35 nm in diameter and displays T = 3 icosahedral symmetry. Thus, 180 copies of VP70 assemble into the immature HAstV particle. The inner layers of immature and mature HAstV particles are nearly identical. In contrast, the outer layers of immature and mature HAstV particles are dramatically different. In immature HAstV particles, the outer layer contains 90 globular spikes. In mature HAstV particles, the outer layer appears to contain only 30 globular spikes located on the icosahedral 2-fold axes. It is not entirely clear whether the other 60 spikes have been released from the virus surface after trypsin digestion, or whether they remain associated with the virus but have become conformationally flexible, resulting in a loss of density upon cryo-EM averaging. It is worth noting that in several reports as well as in our own unpublished studies, there are observations of a significant loss in VP25 upon HAstV virion purification and sodium dodecyl sulfate-polyacrylamide gel electrophoresis (SDS-PAGE) analysis, suggesting that the cryo-EM model showing a loss of 60 spikes may accurately represent the mature, infectious form of HAstV [23,24]. In any case, it is clear that trypsin digestion dramatically modulates the surface of HAstV to increase virus infectivity. Possible mechanisms by which protease maturation increases infectivity, including exposure of a receptor-binding site, promoting virus internalization, or priming the virus for uncoating, have yet to be investigated.

High-resolution information about the structural domains of the astrovirus capsid protein has been obtained by X-ray crystallography. The HAstV capsid core domain (HAstV-1 residues 80–411) forms the T = 3 icosahedral core of the virus particle. The structure of the HAstV capsid core reveals two linear subdomains: a typical jelly-role β-barrel found in many virus capsids (inner core) and a squashed β-barrel (outer core) [14,27] (Figure 3d). Mapping of trypsin cleavage sites onto the structure of the HAstV capsid core domain reveals a model in which multiple cleavage sites may occur on the outer core subdomain, however the majority of proteolytic fragments likely remain bound together through intermolecular interactions in the β-barrel [14]. The HAstV capsid spike domain (residues 430–648) in VP25 and VP27 forms the globular spikes on the outer layer of the virus particle. The structure of the HAstV capsid spike reveals a dimeric protein, where each protomer contains a three-layered β-sandwich fold [14,28,29] (Figure 3e). Previous N-terminal sequencing studies mapped the N-termini of VP27 and VP25 to residues 394 and 424, respectively [11]. This means that VP27 begins before the final β-strand in the core domain, likely resulting in a tethering of the spike domain to the core domain. However, VP25 begins after the core domain in a flexible linker, suggesting that VP25 is either released from the virion or is only weakly associated with it after trypsin digestion. In the context of the entire mature HAstV virion, the observation of spikes only on the icosahedral 2-fold axes, where there are plateaus on the core surface, suggests that the trypsin cleavage site at residue 424 may be inaccessible under these spikes, allowing them to remain tethered as VP27 to the capsid core.

Interestingly, the structure of an avian (turkey) astrovirus capsid spike domain reveals a striking structural divergence compared to the structure of the human astrovirus capsid spike domain [26] (Figure 3f). While both the avian and human capsid spikes contain a β-sandwich fold with similar folding topology, the avian capsid spike contains a number of deletions and structural insertions of helices that set it apart. The differences in structure between the mammalian and avian astrovirus capsid spikes suggest a more ancient evolutionary divergence than anticipated based upon sequence alone.

In all structural studies that have been carried out, there are notable similarities to the capsid of hepatitis E virus (HEV), a member of the *Hepeviridae* family. Like the astrovirus, HEV is an enterically transmitted, nonenveloped, positive-sense, single-stranded RNA virus. There are clear similarities in structural organization of the capsid proteins from the astrovirus and HEV, including positively charged N-termini, conserved inner and outer core domains, variable spike domains, and C-termini that are proteolytically cleaved. As a result, the immature HAstV particle shows a striking resemblance to the T = 3 icosahedral HEV virus-like particle [25] (Figure 3c). Finally, the astrovirus and HEV capsid core domains have high structural homology [14,27], and even the sequence-divergent spike domains contain a related β-sandwich fold [14,26,28,29,30]. Together, the structural similarities between the astrovirus and HEV capsids suggest a much closer phylogenetic relationship between these two virus families than previously realized based upon sequence alone.

## 4. Capsid Interactions with Antibodies

Extracellular astrovirus particles elicit host antibodies that target the astrovirus CP (Figure 1e). Many studies have demonstrated that anti-HAstV antibodies are prevalent in most human serum worldwide [31,32,33,34,35,36,37]. Because astrovirus disease is observed almost exclusively in the young, elderly, and immune-compromised, it is hypothesized that anti-astrovirus antibodies and the adaptive immune response protect healthy adults from reinfection. Consistent with this hypothesis, in two clinical studies of healthy adult volunteers infected with HAstV, individuals with anti-HAstV antibodies had only mild clinical symptoms whereas the few individuals with no detectable anti-HAstV antibodies had more severe disease [35,38]. Further supporting this hypothesis, an immune-compromised patient with a severe and persistent HAstV infection was found to recover following immunoglobulin therapy [39]. Although direct evidence is needed to prove that anti-astrovirus antibodies protect against astrovirus infection, the recent development of a murine astrovirus model of infection provides an exciting new avenue to test this directly [40].

Immunization of mice or rabbits with mature (trypsin-digested) HAstV results in the generation of polyclonal antibodies that can be used to detect HAstV [41,42,43]. Polyclonal antibodies tend to be serotype-specific with little or no cross-reactivity to other HAstV serotypes by immunofluorescence and ELISA. Polyclonal antibodies react by Western blot with all three fragments of the astrovirus capsid protein, VP34 (the CP core) and VP27 and VP25 (the CP spike) [15,23]. ELISA experiments with purified recombinant HAstV CP core and spike domains further confirmed that both regions of the HAstV capsid are antigenic [14]. The CP spike domain had 5–10-fold higher antibody reactivity compared to the CP core, suggesting that the CP spike is the dominant antigen on the HAstV surface [14].

Three studies have reported the generation of anti-HAstV mouse monoclonal antibodies (mAbs) [17,23,41]. Two non-neutralizing antibodies, mAb 8E7 (isotype IgG1) and mAb 8G4 (isotype IgG1), were found to have cross-reactivity to all serotypes tested (HAstV-1-7) by ELISA and immunofluorescence but not Western blot, suggesting these antibodies have a conserved conformation-dependent epitope on the HAstV surface [23,41]. The epitope of mAb 8E7 was narrowed down by deletion analysis to CP residues 71–260 [7], corresponding to the CP inner core subdomain. The high level of sequence conservation on the three-dimensional surface of the CP inner core in the context of the mature T = 3 HAstV model is consistent with the ability of this antibody to recognize several HAstV serotypes [14]. Although mAb 8E7 does not neutralize HAstV infectivity in cell culture, antibodies to this region of the virus may benefit the host through other means such as virus agglutination, phagocytosis of the virus, and/or blocking the ability of the HAstV CP (residues 79–108) to inhibit activation of the host complement system (further discussed below).

Two studies reported the generation of anti-HAstV neutralizing mAbs that neutralize HAstV infectivity in cell culture [17,23]. In one study, three mAbs were capable of neutralizing HAstV in cell culture: mAb 7C2 (isotype IgM) neutralized all serotypes tested (HAstV-1-7) whereas mAb 3B2 (isotype IgG1) and mAb 5B7 (isotype IgG3) neutralized serotypes HAstV-1 and HAstV-7 [23]. All three neutralizing mAbs immunoprecipitate VP25 and/or VP27, revealing that they target the CP spike domain, and competition ELISAs support that the mAbs have overlapping epitopes. Notably, all three mAbs block HAstV attachment to Caco-2 cells (human colon carcinoma cells), suggesting that the mAbs block HAstV infectivity by blocking an essential receptor-binding site on the HAstV CP spike.

**Figure 4:**
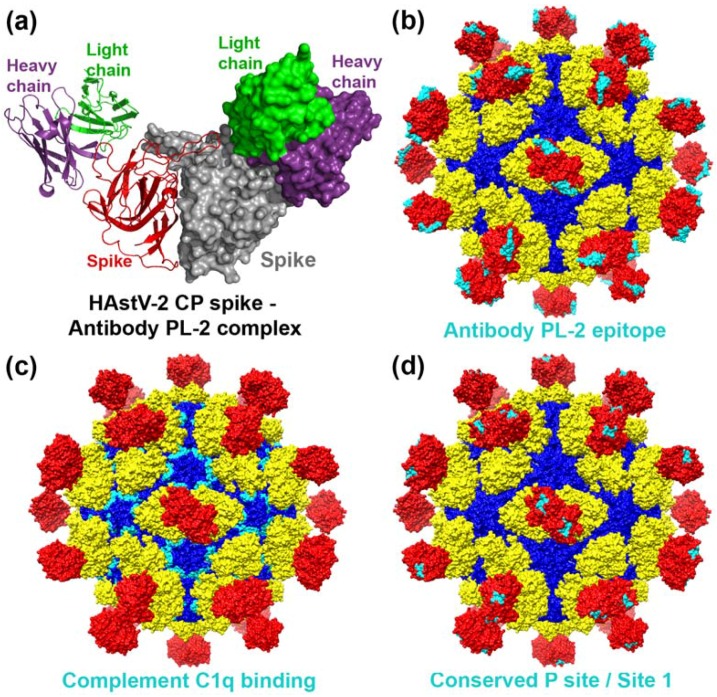
Antibody PL-2 binding site, complement C1q binding site, and putative receptor binding site on the HAstV surface. (**a**) Crystal structure of the HAstV-2 CP spike/scFv PL-2 complex [28]; (**b**) Footprint of the antibody PL-2 epitope (in cyan) on the HAstV-1 surface [28]; (**c**) Location of the complement C1q binding peptide CP1 (CP residues 79–108) (in cyan) on the HAstV-1 surface [45]; (**d**) Location of a putative receptor binding site P site or Site 1 (in cyan) on the HAstV-1 surface [28,29].

Finally, the neutralizing mAb PL-2 (isotype IgG2a) was reported to selectively neutralize serotype HAstV-2 [17]. mAb PL-2 immunoprecipitates VP25 and VP27, revealing that it targets the CP spike domain. De novo sequencing enabled the production of a recombinant single-chain variable fragment (scFv) for antibody PL-2, and recent X-ray crystallographic studies elucidated the structure of the HAstV-2 CP spike-scFv PL-2 complex [28,44] (Figure 4a). The structure reveals a 2:2 binding of two scFv molecules to one CP spike dimer [28]. The epitope of mAb PL-2 is comprised of a conformation-dependent quaternary epitope with many serotype-specific amino acids, consistent with its specificity for serotype HAstV-2 (Figure 4b). However, the epitope also overlaps with several patches of residues that are highly conserved across serotypes HAstV-1-8, suggesting that mAb PL-2 may block an essential site on the HAstV CP spike. The observation that antibody PL-2 blocks attachment of recombinant HAstV-2 CP spike to Caco-2 cells further supports the hypothesis that the HAstV CP spike domain contains an essential receptor-binding site and antibodies neutralize HAstV infectivity by blocking this site [28].

## 5. Capsid Interactions with Complement

Both human and avian astrovirus infection induces limited inflammation and cell death in intestinal tissues, suggesting that astroviruses may disrupt the innate immune system during infection [46,47]. One mechanism by which a virus may disrupt the innate immune system is inhibition of complement activation. Indeed, it was discovered that both the HAstV virus and purified recombinant HAstV CP are able to inhibit serum complement activation [48,49] (Figure 1e). HAstV CP inhibits activation of the classical and lectin pathways but not the alternative pathway. In the classical pathway, CP inhibits C1 activation by binding directly to C1q and displacing the serine protease tetramer C1s-C1r-C1r-C1s [49]. Similarly, in the lectin pathway, CP inhibits mannose-binding lectin (MBL) activation by binding directly to MBL at the same binding site as the serine protease MASP-2.

Analysis of the HAstV CP sequence revealed limited sequence homology between HAstV CP residues 79–139 (located in the CP inner core domain) and human neutrophil defensin-1 (HNP-1), a known inhibitor of C1 and MBL [45]. A peptide containing HAstV CP residues 79–108, called CP1, was found to bind to C1q and compete with binding by full-length recombinant HAstV CP. The CP1 peptide was further found to inhibit C1 and C4 activation and suppress complement activity in serum. Mapping of the CP1 peptide residues onto the HAstV CP core domain structure in the context of the mature T = 3 HAstV model of the virus reveals that part of this peptide (residues 88–100) is exposed on the surface of the virus and may be available for binding to C1q and MBL [14] (Figure 4c). Surprisingly, a derivative of the CP1 peptide called Δ8-22, which has a deletion of residues 86–100 encompassing the surface exposed residues in the CP1 peptide, retains C1q binding activity and inhibits activation of the classical pathway [45]. Thus, further investigation is needed to determine whether the HAstV CP and its peptide derivatives have the same molecular mechanisms of inhibition of complement activation. More importantly, further investigation is needed to understand the role of inhibition of complement activation in astrovirus pathogenesis.

## 6. Virus Entry into Cells

The early interactions of the astrovirus with its host cell have been poorly characterized. The first step in the virus replication cycle is the attachment of the virus capsid to a receptor on the cell surface (Figure 1f). However, the identity of the receptor is not known and it is not known either if different HAstVs share a common receptor or if there are multiple receptors for different serotypes. Also, it has not been determined if there are post-attachment interactions between the virion and cellular co-receptors that are required for virus internalization, as it has been reported for various non-enveloped viruses [50,51,52]. It was observed that HAstVs differ in their efficiencies to replicate in different cell lines, depending on their serotype [53], suggesting that they might use different receptors or co-receptors on the cell membrane during virus entry. However, the existence of post-entry cellular restriction factors that limit virus replication in a serotype-specific manner cannot be excluded. Evidence for cell tropism restriction at the entry level was shown for HAstV-1 in BHK-21 cells (baby hamster kidney cells), which are poorly susceptible to virus infection but become permissive if virus replication is initiated by transfection of in vitro-transcribed viral RNA into the cells [54]. On the other hand, it seems that binding to the cell receptor is not necessarily the interaction that defines the tropism of the virus, since HAstV-8 binds to the surface of MA104 cells (African green monkey kidney cells) only two to three times less efficiently than to the surface of Caco-2 cells (human colon carcinoma cells), while the latter cells are about 1000-fold more permissive to infection than MA104 cells (unpublished results). These findings suggest that after initial binding to the cell surface, the virus has to additionally interact with post attachment cell surface molecules (co-receptors) to enter the cell.

There is evidence that the HAstV CP spike contains a receptor-binding site. Initial evidence came from antigenic and sequence analyses, where neutralizing monoclonal antibodies that immunoprecipitate the CP spike domain (VP25 and VP27) were found to block HAstV attachment to host cells [23]. These findings were confirmed by the recent crystal structure of the HAstV-2 CP spike in complex with the neutralizing antibody scFv PL-2, and by evidence that this scFv inhibits binding of recombinant HAstV CP spike to Caco-2 cells, suggesting that the antibody neutralizes virus infectivity by blocking cell attachment [28]. Mapping the amino acid residues conserved among all eight HAstV serotypes onto the surface of the HAstV CP spike structures reveals several conserved putative receptor-binding sites: (1) The P site/Site 1 is located in a shallow groove on top of the spike and is highly accessible to cell receptors without steric hindrance [28,29] (Figure 4d); (2) the S site/Site 3, located at the side of the spike, which is probably more important for the proper folding of the protein rather than for ligand recognition; (3) an exposed β-turn that forms a knob on each side of the spike; (4) Site 2; and (5) Site 4 [28,29]. Although the putative function, if any, of these conserved regions is not known, they might be involved in the early interactions of the virus with the host cell.

The largest and most surface-exposed putative receptor-binding site, the P site/Site 1, is comprised of several conserved polar and charged amino acids, which are characteristic for carbohydrate binding [29] (Figure 4d). This suggests that, if functional, all HAstV could interact with a common cellular receptor or co-receptor. Of interest, despite the fact that the crystal structure of the TAstV-2 spike shows only distant structural similarities with that of HAstVs, a putative carbohydrate binding site, conserved among members of the avian astrovirus genogroup I, was also described, reinforcing the hypothesis for a role of glycans as cell attachment factors for astroviruses [26]. However, the requirement of carbohydrates for HAstV infection is not clear. The infectivity of HAstV-8 was shown to be only partially blocked by incubation of the virus with heparin, dextran sulfate, or heparin sulfate before infection, and treatment of cells with neuraminidase had no effect on HAstV-8 virus infection, suggesting that sialic acids do not participate in virus–cell interactions [29]. Furthermore, pretreatment of Caco-2 cells with heparinase or chondroitinase did not affect the infectivity of HAstV-1 [29]. In addition, pre-incubation of HAstV-1, HAstV-2, or HAstV-8 with diverse complex milk fractions rich in polysaccharides before infection had a limited effect on virus infectivity, while these milk fractions sharply inhibited the infectivity and cell binding of different rotavirus strains, which are known to require glycans for cell attachment [55]. Finally, binding studies of recombinant HAstV-1 CP spike and turkey astrovirus 2 CP spike in glycan arrays at both neutral and acidic pH showed no significant binding [56]. Thus, these data suggest that despite the indication of a carbohydrate binding site on the HAstV CP spike, there is little evidence to support a major role for glycan receptors in HAstV infection.

Mature (trypsin-digested) HAstV-8 has been estimated to attach to cultured Caco-2 cells with a half-time of approximately 10 min [57]. After binding to the cell surface, the virus particles are internalized by a clathrin-dependent endocytic process (Figure 1f). Early studies on HAstV-1 entry in HEK293 cells demonstrated the presence of virions in coated pits and coated vesicles and the requirement for endosome acidification, indicating endocytosis as the most likely mechanism for HAstV entry [58]. Recently, clathrin-dependent endocytosis was confirmed to be a functional pathway for the entry of HAstV into Caco-2 cells [57]. Drugs that affect clathrin assembly, endosome acidification, and dynamics of actin filaments, as well as those that decrease cellular cholesterol and interfere with vesicular transport, reduced the infectivity of HAstV-8. The infectivity of HAstV was also reduced by knocking down the expression of the clathrin heavy chain by RNA interference and by expression of dominant-negative mutants of dynamin [57]. After cell internalization, an uncoating event must occur to deliver the virus genome into the cytosol to start viral protein synthesis (Figure 1g). The observation that HAstV infectivity is reduced by drugs that raise the intracellular pH or by knocking down the expression of Rab7 suggests that endosome maturation has to occur and that the virus has to arrive to late endosomes to uncoat, an event that occurs with a half-time of approximately 130 min [57]. Still, the mechanism through which the astrovirus genome is released from the infecting virus particle, the specific cell site where it occurs, and the cellular and viral factors involved in these events are unknown.

The early interaction of HAstV with the host cell induces the activation of the extracellular signal-regulated kinase (ERK1/2) and the phosphoinositide 3-kinase (PI3K) pathways [59,60] (Figure 1g). HAstVs induce the transient phosphorylation of ERK1/2 within the first 15 min after virus attachment, independently of virus replication, since infectious and ultraviolet light -inactivated HAstV activate the pathway with the same kinetics, suggesting that this cascade is triggered during virus binding or entry into the cell [59]. This conclusion is supported by the observation that the activation of ERK1/2 is required early during infection to establish a productive virus replication cycle, and inhibitors of this kinase block the early synthesis of viral proteins from the incoming viral genomic RNAs, with the consequent reduction of late viral protein and RNA synthesis, and reduced virus yield [59]. On the other hand, the activation of the PI3K-mediated cascade was also shown to occur at early times of HAstV-1 infection, as judged by the timeframe of Akt phosphorylation, and this pathway seems to act independently of that mediated by ERK1/2 [60]. The viral factors triggering the activation of these two pathways as well as the mechanism through which they facilitate virus entry and replication are not known.

One of the hurdles to significant progress in our understanding of early interactions of the astrovirus with host cells has been the difficulty to obtain sufficient amounts of purified virus [11,22], however several recently developed tools may stimulate advancements in this area. First, a valuable tool to advance the characterization of the early interactions of the virus with the cell, including cell receptor identification, is the ability to produce large amounts of recombinant astrovirus CP spike that folds into a structure indistinguishable from that of the spike on viral particles, as judged by their ability to form dimers, interact with neutralizing antibodies, and bind to the cell surface [14,26,28,29]. In addition, the ability to produce large amounts of recombinant astrovirus CP core will also allow for experiments to test for potential interactions with host cell factors [14,27]. Another important tool to study initial virus–cell interactions is virus-like particles (VLPs) generated by the expression of recombinant full-length CP in insect cells using the baculovirus expression vector system [61]. Like infectious HAstV and UV-inactivated HAstV, VLPs interact with polarized Caco-2 cell monolayers, disrupting the actin cytoskeleton and tight junction complex, leading to increased epithelial barrier permeability. These events, although independent of virus replication, are detected 20 h post-infection or after addition of VLPs, indicating that they are not likely related to virus entry [62]. Even more striking, turkey astrovirus 2 VLPs, like the infectious virus, reproduce in vivo the observations made in Caco-2 cells, increasing the intestinal barrier permeability and inducing diarrhea in turkey poults when administered orally [63]. Together, these data provide interesting findings that the astrovirus CP may play a role in disruption of tight junctions, increased intestinal barrier permeability, and disease.

## 7. Conclusions

Overall, recent studies on the astrovirus CP have unveiled exciting new insights into the virus structure, proteolytic maturation and infectivity, interactions with host antibodies and complement, and entry into host cells. These studies have advanced our understanding of the astrovirus life cycle and have afforded new tools and methods to study the virus. These studies have major implications for the development of therapies to prevent and treat astrovirus infection. Nevertheless, there are still many significant questions that remain unanswered, including:

Capsid assembly and proteolytic processing:

- What is the role of the acidic region at the C-terminus of the CP?
- What is the mechanism of astrovirus release from the host cell?
- Which extracellular protease(s) play a role in HAstV maturation in vivo?
- What is the mechanism by which protease maturation increases HAstV infectivity?
- Which of the protease cleavage sites are essential for HAstV infectivity?

Capsid structure:

- Are the spike dimers homodimeric for VP27 or heterodimeric for VP25 and VP27?
- What are the structures of CPs from other sequence-divergent astroviruses?
- What are the high-resolution structures of the immature and mature astrovirus particles?

Capsid interactions with antibodies and complement:

- Where do other neutralizing and non-neutralizing antibodies bind?
- What are the mechanisms by which neutralizing antibodies block infection?
- Do antibodies protect from astrovirus infection in vivo?
- What is the role of CP inhibition of complement activation in astrovirus pathogenesis?

Virus entry into cells:

- What is the astrovirus host cell receptor and do astroviruses use co-receptors?
- Do glycans play a role in virus cell entry?
- Where are the receptor binding site(s) on the CP surface?
- What viral factors trigger activation of the ERK1/2 and PI3K pathways and how do they facilitate virus entry and replication?
- How and where is the astrovirus genome released into the cytoplasm?
- How does the CP modulate tight junctions and intestinal barrier permeability?
